# Occurrence of impaired swallowing ability and change over a year in older adults living in nursing homes

**DOI:** 10.1038/s41598-025-19729-6

**Published:** 2025-09-17

**Authors:** Ida Crossler, Clara Shrestha Jensen, Karin Eriksson, Lisa Tuomi

**Affiliations:** 1 Department of Health and Rehabilitation, Institute of Neuroscience and Physiology, Sahlgrenska Academy, University of Gothenburg, Gothenburg, Sweden; 2 Region Västra Götaland, Department of Neurology, Sahlgrenska University Hospital, Gothenburg, Sweden; 3 Region Västra Götaland, Department of Otorhinolaryngology, Head and Neck Surgery, Sahlgrenska University Hospital, Gothenburg, Sweden

**Keywords:** Gugging swallowing screen, Dysphagia, Swallowing, Nursing homes, Screening, Geriatrics, Nutrition, Digestive signs and symptoms

## Abstract

The aim of the present study was to investigate the prevalence of dysphagia in three nursing homes and to investigate whether swallowing function changes over a year. Seventy-three individuals participated (median age 89 years) and were tested with the Gugging Swallowing Screen (GUSS). The participants estimated self-perceived swallowing ability with a self-report questionnaire. Thirty-eight participants (52%) exhibited dysphagia according to the GUSS. Thirty-six participants (49%) showed symptoms of dysphagia according to the self-report questionnaire. The study also investigated how well the results from the GUSS agreed with self-rated swallowing function and found 60% agreement. No association was found between age and swallowing function. Twenty participants were examined twice, one year apart with no statistically significant change between these occasions. Results show that approximately half of the participants have persistent swallowing difficulties after one year. Swallowing difficulties are common in older adults living in nursing homes, even though few have an ongoing intervention to improve swallowing safety and efficiency.

## Introduction

Dysphagia, or difficulty swallowing, encompass unsafe and ineffective transport of food and liquid from the mouth to the stomach^1^. It can occur as a result of neurological, structural or functional impairments, leading to an increased risk of malnutrition, dehydration and aspiration pneumonia^2^. Further, swallowing may be affected by age-related physiological changes, such as muscle atrophy, increased stiffness, reduced sensation, slower reflexes, reduced saliva production and reduced taste and smell, resulting in a delayed initiation of the swallowing process, longer duration of the upper esophageal sphincter opening and an increased risk of oral and pharyngeal residue, with reduced airway protection^1,3–8^. These age-related changes in combination with commonly occurring comorbidities, may manifest as disordered swallowing, i.e. dysphagia^9^. International studies report that approximately 10–30% of people over 65 years of age have some form of swallowing difficulty^10–14^. In older adults living in nursing homes, the prevalence is even higher, up to 53%^15–20^. Despite its prevalence, dysphagia is underdiagnosed, and many do not receive treatment^11^.

To the authors’ knowledge, the development of swallowing dysfunction is not well documented through longitudinal studies. However, one longitudinal study investigated self-rated symptoms of dysphagia in a large group of older people (> 50 years) from the UK population between 2009 and 2012^21^. Over these three years, there was no change in self-rated swallowing function. Further, it has been highlighted that there is a need for systematic assessment of dysphagia in order to provide accurate diagnosis and timely treatment followed by regular assessments to capture possible changes, to reduce the risk for complications such as pneumonia^10^. To investigate changes over time, and to establish whether a swallowing screening tool may capture these changes, more longitudinal studies of dysphagia in older people are needed.

Rivelsrud et al. describe in their systematic review on the prevalence of dysphagia in different healthcare settings, that the general knowledge of dysphagia and its symptoms among healthcare professionals is lacking, and that procedures for identifying dysphagia are insufficient^9^. Detecting dysphagia at an early stage is a crucial factor in providing effective treatment and reducing the risk of adverse health effects in the older person^16^. Healthcare professionals, such as the nurses, assistant nurses and nursing assistants working closely with residents are the ones who can identify and prevent dysphagia complications at an early stage^17^. For healthcare professionals to provide residents with the right adaptations, it is important to be able to identify the presence of dysphagia and gain more knowledge about symptoms of dysphagia. Using swallowing screening allows staff to quickly recognize whether the person has signs of swallowing difficulties, prompting a referral to a speech-language pathologist (SLP) fort further assessment and thereafter, possible intervention to improve swallowing function. By identifying and ultimately intervening with further referral and possible swallowing intervention to those who show signs of swallowing difficulties, the well-being of the population can be improved. Two reviews have found that the Gugging Swallowing Screen (GUSS) is suitable for the identification of signs of dysphagia^2,22^ and that it can also be administered by nurses^22^. However, more studies are needed to investigate how to implement the use of routine screening of swallowing difficulties, in nursing homes with limited resources and staff.

The overall aim of the study was to investigate and describe the prevalence of impaired swallowing ability in older adults living in nursing homes and to investigate whether swallowing function changes over a year.

## Material and methods

**Fig. 1:**
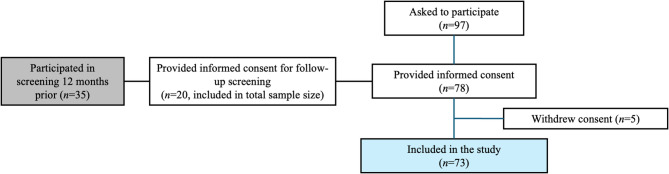
Flow chart demonstrating participant inclusion.

Older adults living in three different nursing homes in Gothenburg, Sweden, were asked about participation in the present study. Inclusion criteria were age > 65 years, living in one of three nursing homes, and cognitive function and knowledge of the Swedish language sufficient to be able to provide informed consent. Participants who could not eat or drink orally were excluded. The nursing staff helped to identify possible participants in the study, i.e. who met the inclusion criteria. They also helped to provide information about the current nutritional status. To evaluate if there was any detectable change in swallowing function over 12 months, the same participants that were included in the study preceding the current one by Engberg et al.^23^ were asked to participate in a follow-up testing 12 months after their first testing. Of the 35 participants in the study by Engberg et al., 20 could be included in the present study, using the same inclusion criteria as in the present study. During the first testing, they all underwent screening with the GUSS and 4QT. Reasons for not being included in the present were that they declined participation, did not live at the nursing home anymore, had reduced general condition, or were deceased. A total of 73 participants were included in the present study (Fig. 1).

Information about participant demography was registered in collaboration with the nursing home staff, using information found in their medical chart. This included information about age, sex, medical history possibly connected to swallowing difficulties as stated in Table 1, use of nutritional supplements and use of texture modifications.

**Table 1:** Descriptive characteristics of study participants.

		Total (*N =* 73)	Participants screened twice (*n* = 20)
Age (years)	Median (min-max)	89 (68–102) *n* (%)	86 (68–101) *n* (%)
Sex	Female	56 (77%)	13 (65%)
	Male	17 (23%)	7 (35%)
Medical background with risk for dysphagia and/or malnutrition	Stroke	25 (34%)	7 (35%)
	Parkinsons disease	4 (5%)	1 (5%)
	Heart failure	15 (21%)	2 (10%)
	Renal failure	10 (14%)	2 (10%)
	Diabetes mellitus	14 (19%)	2 (10%)
	Atrial fibrillation	26 (36%)	5 (25%)
	Cancer^a^	13 (18%)	5 (25%)
	Asthma	7 (10%)	2 (10%)
	Chronic obstructive	5 (7%)	3 (15%)
	Pulmonary disease depression	13 (18%)	4 (20%)
Dietary customization	Nutritional supplement	12 (16%)	2 (10%)
	Texture modification ^b^	13 (18%)	6 (30%)

^a^Different cancer sites such as breast, bladder, skin, corpus uteri, small intestine, ovaries, bile duct, prostate.

^b^Texture modification included thickened liquids and puréed food.

### Gugging swallowing screen (GUSS)

The Swedish version of the GUSS was used to screen the swallowing function^24^. It includes the assessment of risk of aspiration through swallowing of different consistencies, and results in different recommendations about texture modifications and further evaluation of swallow function. The GUSS was originally developed to identify the prevalence of dysphagia following stroke^25^, but is highly relevant for use in older adults as well^2,23^. The screening instrument has been found valid, showing high sensitivity and specificity^26–28^. The instrument has been validated into several languages^26,29^. The GUSS has been evaluated in a large healty older population in Turkey, and was found valid and reliable, with high sensitivity (95.5%) and specificity (94.4%)^30^. GUSS has also been translated into Swedish and found valid and reliable^24^however not yet validated in a mixed population of older adults.

The screening instrument consists of two parts. The first part examines indirect swallowing ability, and the second part examines direct swallowing ability with three different consistencies, performed sequentially^25^. The consistencies are labelled according to the International Dysphagia Diet Standardisation Initiative (IDDSI) framework^30^. In the first part, the following is observed: level of alertness, ability to cough, swallowing of saliva, presence of drooling and voice changes. In this part, the participant can receive 1 point (yes) or 0 points (no) on each item. In part two, swallowing ability is assessed by having the participant swallow moderately thick (IDDSI level 3) and thin liquid (IDDSI level 0) in increasing amounts and solid food (IDDSI level 7). If any of the following occur during swallowing: failure to swallow, slow swallowing, successful swallowing, coughing during swallowing, drooling during swallowing and change in voice quality after swallowing this is given different scores. Under the first assessment parameter ‘Swallowing’, the subject can be given 0 points (no swallowing) 1 point (delayed swallowing) or 2 points (successful swallowing), then the other assessment parameters ‘coughing’, ‘drooling’ and ‘change in voice’ can be given either 0 points (yes) or 1 point (no). In case of less than 5 points in part 1 or in any of the consistencies on Part 2, the screening is concluded.

The subtest scores are totaled and the lower the score, the more severe the swallowing difficulty, leading to recommendations such as consistency adjustment. The total score on the GUSS corresponds to different levels of severity of swallowing difficulty. 20 points correspond to normal swallowing, 15–19 points correspond to mild dysphagia with aspiration risk, if the participant scores between 10 and 14 points it corresponds to moderate dysphagia with aspiration risk and if the participant scores between 0 and 9 points it corresponds to severe dysphagia with high aspiration risk. In cases of mild-severe dysphagia recommendations include different texture modifications and referral to speech-language pathologists (SLP) for instrumental assessments of swallowing function. In case of a failed screen, nursing staff were informed and a referral to the SLP was made with the participant’s consent. However, the possible follow-up with an SLP was not included in the study.

In the present study the GUSS was administered by authors I.C and C.SJ (fourth-year SLP students at the time) according to the procedure described in the original publication and the Swedish translation and validation study^24,25^. Before testing started the testers completed a half-day training of using the instrument with an SLP with > 20 years of experience as well as partaking in a practical training session for nursing staff at the nursing home. For 15% of the participants both testers completed the GUSS simultaneously and independently from each other, to allow for inter-rater reliability calculations.

### Questions about swallowing

The participants were asked to rate their perceived swallowing problems according to four questions, the 4QT^31^. The questions concern coughing while eating or drinking, prolonged meal duration, change in diet/texture and voice change in connection to eating or drinking. The questions are asked in a yes/no manner, and each “yes” is given a score of 1, and responses are summed. When 4QT is used, a score of 1 or more is considered having a high risk of dysphagia, needing further assessment^31^. The 4QT was recently validated into Danish in a cohort of people above 65 years of age^32^. It demonstrated high sensitivity (84%) but low specificity (36%) and low reliability (Cronbach’s alpha 0.58). The Swedish version has not been validated.

### Ethical considerations

The study was conducted in accordance to the Declaration of Helsinki and was approved by the Swedish Ethical Review Authority, reference number 2022-03960-0, date of approval 2022-09-07. All participants provided their informed consent prior to inclusion in the study.

### Statistical analyses

Data analyses were performed using IBM SPSS Statistics 28.0.1.1 software. Significance level was set at 0.05. Data were not expected to be normally distributed, therefore non-parametric statistics were used. To answer the question regarding the prevalence of dysphagia, the total number of participants showing abnormality on GUSS was calculated, divided by the total number of participants, the same regarding the prevalence of dysphagia with 4QT, and the prevalence is presented as the prevalence and 95% confidence intervals. Participants were classified according to whether they had dysphagia according to GUSS (< 20 points) or 4QT (> 0 points). The agreement between the two was calculated based on the percentage of exact agreement between the two assessment instruments. Spearman’s rank correlation was used to answer the question of whether there was a relationship between swallowing function and age.

To answer the question of whether swallowing function changes over one year as measured by GUSS, data were analyzed using the Wilcoxon signed-rank test.

For the inter-rater reliability of the GUSS assessments percent point-by-point agreement was calculated on 15% (*n* = 11) of the total screenings. These assessments were performed by the two authors simultaneously and independently from each other.

## Results

A total of 73 participants were included in the present study. Their mean age was 89 years, and 77% of participants were women. Thirteen participants (18%) had some texture modification to facilitate swallowing. The medical background showed that 34% of participants had a stroke and 5% were diagnosed with Parkinson’s disease. Further details of the participants are found in Table 1.

The prevalence of swallowing difficulties according to the GUSS is found in Table 2. A total of 38 participants (52%) demonstrated dysphagia of any level, where 27 participants (37%) demonstrated moderate-severe dysphagia. All participants passed part 1 of the GUSS.

**Table 2:** The prevalence of dysphagia according to the gugging swallowing screen (GUSS) and self-perceived swallowing difficulties.

	*n* (%)	95% confidence interval
Dysphagia classification according to the GUSS		
No dysphagia (20 points)	35 (48%)	36–60%
Mild dysphagia (15–19 points)	11 (15%)	8–25%
Moderate dysphagia (10–14 points)	16 (22%)	13–33%
Severe dysphagia (0–9 points)	11 (15%)	8–25%
Dysphagia according to questions of swallowing in the 4QT	36 (49%)	37–61%

Inter-rater reliability was calculated in 15% of the assessments and resulted in 100% inter-rater reliability.

Similar numbers regarding prevalence of dysphagia were found when evaluating through the 4QT, where 36 (49%) of participants demonstrated signs of dysphagia. For 44 of the participants, the classification of dysphagia was in agreement between the GUSS and 4QT, i.e. resulting in either a pass or fail on the screening for both screening methods, resulting in a 60% agreement between the assessment methods.

A Spearman’s rank-order correlation was conducted to examine the relationship between age and swallowing function measured with the GUSS. The correlation was negative and not statistically significant, r_s_ = − 0.203, *p* = .085, indicating no significant association between the variables.

Swallowing function was evaluated twice with approximately 12 months in between for a subset of participants (*n* = 20). At the first occasion, 8 of them (40%) were found to have dysphagia. At the follow-up occasion, 12 of them (65%) demonstrated dysphagia. There was no statistically significant difference within the group, however, individual differences were found, where 10 deteriorated (50%), 6 participants (30%) demonstrated no change, and 4 (20%) demonstrated improvement (*Z* = 36, *p* = .29.).

## Discussion

The aim of this study was to identify the prevalence of dysphagia measured by GUSS and self-assessment of symptoms of swallowing difficulties in residents of nursing homes. Furthermore, the study also aimed to investigate whether swallowing function changes over a year. The prevalence of dysphagia measured with the GUSS showed that 52% of the participants had some form of dysphagia. Participants’ self-reported scores showed that just under half of the participants experienced symptoms of swallowing difficulties. Regarding change in swallowing function over one year, it was found that the swallowing ability of 50% had deteriorated, some had improved and in some participants the ability was unchanged. This may be because the present study had too short a time span between screening sessions to show any significant changes.

A systematic review^9^ showed that the prevalence of dysphagia was highest in nursing homes (50%) of the different care settings compared, as expected, since the populations in the nursing homes were older, and bore more medical conditions associated with dysphagia. In the present study, the prevalence of dysphagia was reported in 52%, confirming that older people in nursing homes are a risk group for dysphagia. In total, 13 participants had some form of dietary adjustment in the past and the results showed that over half had some form of impaired swallowing ability. This could be speculated to possibly indicate that the awareness of swallowing difficulties is low for people living in nursing homes further indicating the need for screening to identify dysphagia. Several studies, evaluating the prevalence of dysphagia in older adults living in nursing facilities have demonstrated similar dysphagia prevalence^17,18^. However, a recent study investigating the prevalence of dysphagia in older adults using GUSS found a lower prevalence of 31%^3^. A possible explanation for the differing prevalence numbers may be that the present study allowed inclusion of participants with some cognitive impairment, however sufficient cognition to independently consent to study participation, unlike Engberg et al.^23^ who only included participants without cognitive impairment.

Previously, it has been demonstrated that older people regardless of cognitive function have difficulty self-assessing their swallowing ability^33^. Therefore, it is important to investigate with both direct and indirect screening also in people with cognitive impairment. The self-assessment resulted in a similar prevalence rate as screening, which can be seen as a sign that the 4QT is a form that can measure the presence of dysphagia. However, the agreement between the GUSS and the self-assessment was only moderate (60%), which rather indicates that they measure slightly different symptoms and complement each other. There are several studies that have investigated the prevalence of dysphagia in older adults using only indirect methods in the form of self-assessment questionnaires or questionnaires completed by carers. These studies have shown a lower prevalence rate of dysphagia (12–15%)^15,16,19^. This may be related to the fact that older people have difficulties understanding their symptoms and what they mean.

The present study found no statistically significant association between swallowing function and age. One explanation for this lack of association might be due to the lack of younger individuals, since the median age was 89 years. If the sample would have included younger individuals as well, the results might have been different. One other possible explanation for this may be that age per se does not cause dysphagia in the individual. The older adult population living in nursing homes is a group at risk of dehydration, malnutrition, pneumonia and aspiration due to multiple illnesses. With increasing age, comorbidity also becomes more common, where medical conditions may be associated with dysphagia^9^. To improve knowledge about the signs that characterize swallowing difficulties, how to identify it and what to do about it nursing staff need support and tools for systematic screening of swallowing dysfunction.

Swallowing function is known to deteriorate with increased age. However, there are few longitudinal studies that investigate how swallowing function changes within the same individuals over time. Nimmons et al.^21^ investigated whether swallowing function changes over time and included a population group (> 50 years) with a median age of 81 years. What was seen in the present study was that participants’ swallowing function improved, deteriorated or remained unchanged over 12 month’s time as measured by the GUSS, but no statistically significant change was found, even though 50% deteriorated. It is possible that 12 months is too short a time to capture any longitudinal changes and that any differences could be found if follow-up was done over a longer period, however, it is more likely that the sample size was too small to detect any changes. The result showed that 50% of the participants did deteriorate, which is a large change, even though that it did not result in a statistically significant result. In the study by Nimmons et al., participants were followed for a longer period, 3 years, and still showed similar results^21^. A further explanation for the lack of significant change at the group level may be that those who were most alert and healthy were also those who wanted to participate again, while those who had deteriorated in general condition or died could not participate. Since half of the participant did show deteriorating function, it is important to examine swallowing ability to capture change over time, and to revise given recommendations when necessary.

There are studies that highlight the importance of using validated screening instruments for groups at high risk of dysphagia, including older adults^12,16,34^. Early identification of people at risk of dysphagia is crucial to reduce the health risks that dysphagia can lead to. Furthermore, Speyer et al. discuss that training in dysphagia screening and assessment should be provided for doctors and nurses working with people where dysphagia is common^34^. It would be useful to have access to a screening tool that is easy for residential staff to perform and GUSS is just such an instrument^16^. With the GUSS, several different consistencies are used for screening, which is an advantage in order to be able to see how residents ingest other consistencies than just thin liquid which is what is most commonly assessed^17^. It provides recommendation on texture modifications based on GUSS test results, thereby reducing the risk of patients going without food while waiting for an SLP assessment.

A limitation of the study may be the lack of standardized assessment of cognitive function prior to inclusion, possible resulting in different interpretation of the inclusion criteria by different nurses, and possibly skewing the results, since swallowing difficulties may be more common in participants with more profound cognitive impairment. Due to the small number of participants, results should be interpreted with caution. More studies are needed with larger numbers of participants to investigate how to implement the use of dysphagia screening in a systematic and routine way in nursing homes, which have limited resources and staff.

## Conclusion

Dysphagia was found in approximately half of the participants in the present study, even though only a few had known difficulties before inclusion in the study. This is in line with previous research. The GUSS seem to be able to capture individual changes in swallowing function, even though no statistically significant difference was found over 12 months. The study highlights the need to increase detection of dysphagia and symptoms of dysphagia through regular screening within the nursing home setting.

## Data Availability

The data that support the findings of this study are not openly available due to reasons of sensitivity. Data are located in controlled access data storage at Sahlgrenska Academy. For questions regarding data, please contact corresponding author Lisa Tuomi.
